# Incoherence in the Brain Death Guideline Regarding Brain Blood Flow Testing: Lessons from the Much-Publicized Case of Zack Dunlap

**DOI:** 10.1177/00243639251317690

**Published:** 2025-02-04

**Authors:** Doyen Nguyen, Christine M. Zainer

**Affiliations:** 1 Institute of Bioethics, 59207 Universidade Católica Portuguesa, Lisboa, Portugal; 2 Anesthesiology, 5506 Medical College of Wisconsin, Milwaukee, WI, USA

**Keywords:** Brain death determination guideline, brain blood flow scintigraphy, traumatic brain injury, cognitive-motor dissociation, covert consciousness

## Abstract

At age 21, following a severe traumatic brain injury, Zack Dunlap was declared brain-dead according to the American Academy of Neurology guideline (Guideline) when he met the clinical criteria of brain death (minus apnea testing because of bradycardia) with technetium-99m diethylene-triamine-pentaacetate scintigraphy reported as showing no intracranial blood flow. His parents agreed to organ donation. During preparations for organ donation, Zack manifested a purposeful movement in response to a noxious stimulus made by his cousin. Following subsequent neurological recovery, he has returned to a normal life, holding steady employment and raising a family. During an interview, he reported that while in coma, he heard a doctor say that he was brain-dead and felt angry about it. His experience fits the phenomenon of cognitive-motor dissociation. Recently, Zack's medical records were made available to the first author. A critical review of the records uncovered a problem inherent in the logic of the Guideline algorithm regarding brain blood flow scintigraphy. This article discusses the lessons drawn from Zack's case, namely, that both the aforementioned problem and the occurrence of cognitive-motor dissociation in patients deemed to be brain-dead can pose a significant risk of a false-positive declaration of death.

The determination of brain death/death by neurologic criteria (BD/DNC) in adults in the United States is based on the American Academy of Neurology (AAN) guideline (henceforth, the Guideline) issued in 1995 (Wijdicks 1995) with subsequent updates in 2010 (Wijdicks et al. 2010) and 2023 (Greer et al. 2023). While BD/DNC protocols in many European countries require ancillary testing for brain blood flow (BBF) and/or brain electrical activity (Citerio et al. 2014), such testing is optional in US institutions. The Guideline emphasizes that BD/DNC is a clinical diagnosis requiring only a bedside neurological examination. However, if the full battery of bedside clinical tests cannot be completed, namely, when the apnea test cannot be performed or has to be aborted because of safety reasons, BD/DNC can still be diagnosed based on ancillary tests, especially BBF studies. The case of Zack Dunlap, who in 2007 had a technetium-99m diethylene-triamine-pentaacetate (Tc99m-DTPA) scintigraphy performed, belongs to this category. Zack's case has been much publicized as one with full recovery following a BD/DNC diagnosis (Celizic 2008; Morales 2008).

In late 2023, the first author received Zack's medical records from the second author. Supplementing the records are the statements of Zack and his cousin during a TV interview in March 2008 (Morales 2008). A critical review of the records revealed that this was an unusually difficult case, one that brings into focus two challenging issues in BD/DNC diagnosis: (i) the specificity and confirmatory value of BBF scintigraphy, and (ii) the condition of cognitive-motor dissociation (CMD) in the acute phase of brain injury.

## Case History

On November 17, 2007, at age 21, Zack sustained a severe head injury in a crash accident while driving an all-terrain vehicle without a helmet. Rushed by ambulance to a local emergency room in Frederick, Oklahoma, he was found to be combative with a Glasgow Coma Scale (GCS) score of 6. He received intravenously 7.5 mg of midazolam, 12.5 gm of mannitol, 12 mg of dexamethasone, 1 gm of ceftriaxone, tetanus toxoid, and vecuronium. He was ventilated and airlifted to a level II regional trauma center in Wichita Falls, Texas.

On admission at the regional trauma center, Zack's blood pressure was 223/118 and his heart rate was 87. He received 20 mg of labetalol intravenously and a propofol infusion which decreased the blood pressure to 170/126 with a heart rate of 80. He was unresponsive, not overbreathing the ventilator, with GCS 3, pupils at 2 mm and sluggish, and cerebrospinal fluid (CSF) otorrhea from the right ear. He was placed on the traumatic brain injury (TBI) protocol which included 30-degree head elevation, sedation with propofol infusion, mannitol osmotic treatment, and prophylactic antibiotics.

**Figure 1.**
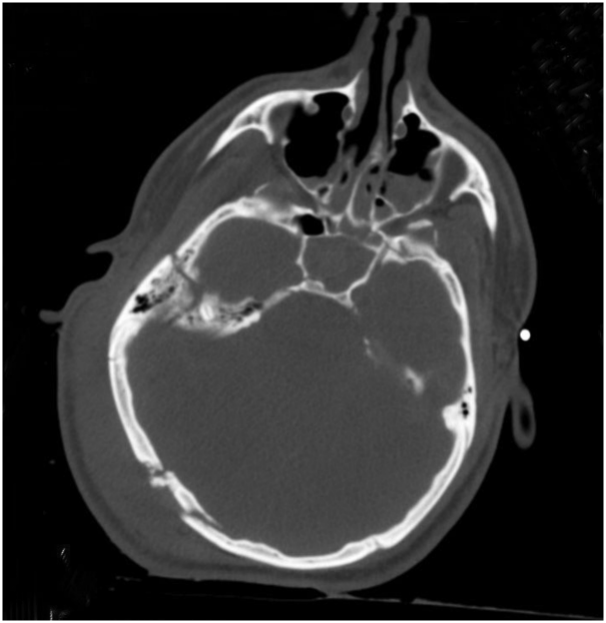
Computerized tomography scan of the head on admission (1.8 h after injury). There are basilar skull fractures involving the right posterior parietal occipital region with a depressed large fragment and extension of the fracture lines into the right temporal bone involving the right mastoid and middle ear structures.

**Figure 2.**
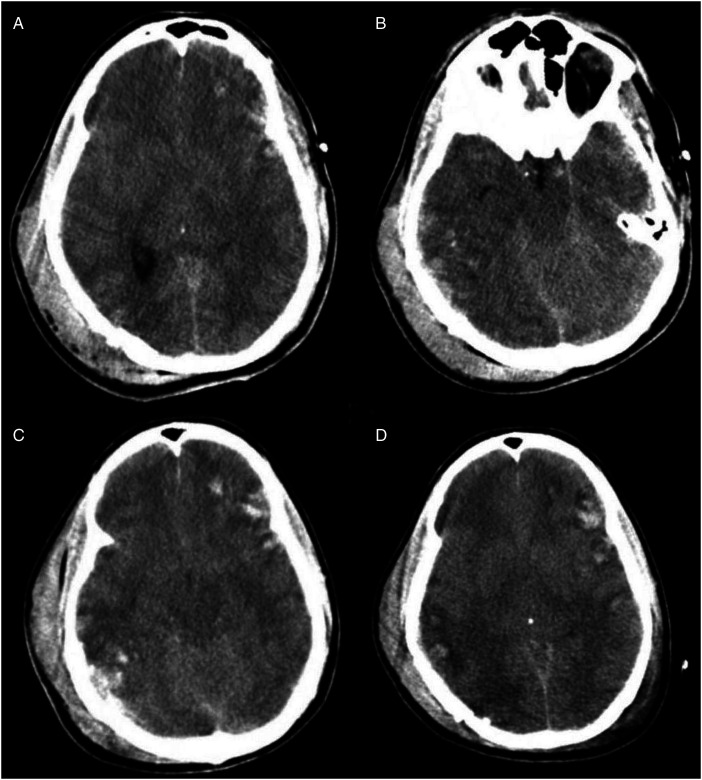
Four views from computerized tomography (CT) scans of the head from 1.8 h after injury to 44.7 h after injury. At 1.8 h, there are hemorrhagic contusions in the right temporal and posterior parietal regions, as well as in the left frontal and temporoparietal regions (A, B). Similar findings are present on follow-up CT scans at 12.9 h (C) and 44.7 h (D).

Computed tomography (CT) scan of the head, obtained around 1.8 h (the timing of events will be presented as hours from the time of injury), showed basilar skull fractures of the right posterior parietal occipital region with a depressed large fragment and extension of the fracture lines into the right temporal bone involving the right mastoid and middle ear structures (Figure 1). The fracture lines also extended into the right lateral frontal bone. The brain showed generalized cerebral edema with effacement of sulci and gyri, parenchymal hemorrhages in the right temporal and posterior parietal regions, and the left frontal and temporoparietal regions (Figure 2A and B). The ventricles were normal in size with no evidence of hydrocephalus or midline shift. Computed tomography scan of the spine, chest, and abdomen showed a fractured right clavicle and no other abnormalities. Follow-up CT scans of the head at 12.9 and 44.7 h showed findings similar to those on admission (Figure 2C and D).

**Figure 3.**
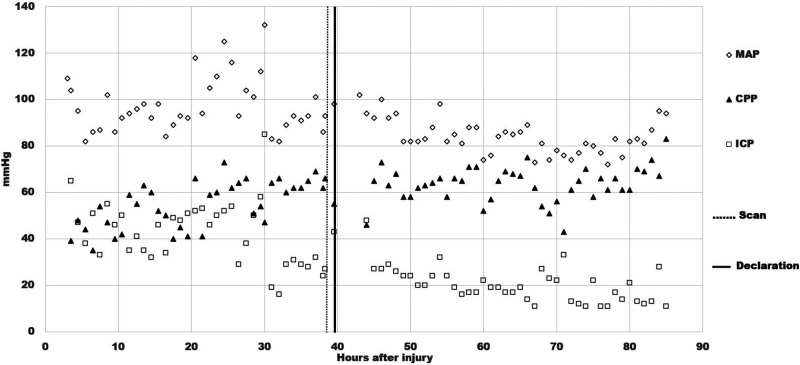
Time courses of mean arterial pressure (MAP), intracranial pressure (ICP), and cerebral perfusion pressure (CPP), together with the timing of the radionuclide blood flow scan and the declaration of brain death.

An intracranial pressure (ICP) monitoring device was inserted around 4 h; the immediate ICP reading was 60+ mmHg. Because of signs of hemodynamic instability, phenylephrine was started around 11.5 h to increase mean arterial pressure (MAP) and keep cerebral perfusion pressure (CPP) above 70. The ICP remained elevated, above 30 mmHg despite osmotic treatment (Figure 3). From around 18 h, Zack's condition worsened with sustained intracranial hypertension into the 50 mmHg range. Around 30 h, the ICP spiked to 85 mmHg, with tachycardia in the 160 range and systolic blood pressure of 220–240 mmHg. Both pupils were nonreactive, 6 mm on the right, and irregular 5–6 mm on the left. Because of clinical deterioration, Zack underwent a Tc99m-DTPA scintigraphy around 38.6 h. The radiologist reported no evidence of BBF on the perfusion phase images and mentioned nothing about the venous sinuses on the delayed blood pool phase images.

**Figure 4.**
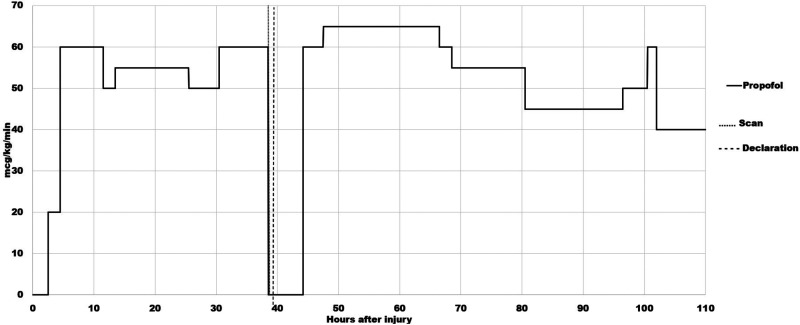
Time course of propofol infusion, together with the timing of the radionuclide blood flow scan and of the declaration of brain death. The propofol infusion was started at 2.5 h after the injury at the rate of 20 mcg/kg/min. The dose was increased to 60 mcg/kg/min at 4.5 h. Thereafter, the rate of propofol infusion varied between 50 mcg/kg/min and 60 mcg/kg/min until 38.5 h when the infusion was stopped. Propofol infusion was restarted at 44.2 h at the rate of 60 mcg/kg/min; the dose was increased to 65 mcg/kg/min at 47.5 h and maintained at this level until 66.5 h when the dose was reduced to 60 mcg/kg/min, and further reduced to 55 mcg at 68.5 h. At 80.5 h, the dose was decreased 45 mcg/kg/min. At 102.5 h, the dose was reduced to 40 mcg/kg/min. The propofol infusion was maintained at this level for another 65 hours. Thereafter, the patient was gradually weaned off propofol, and the infusion was discontinued 14 hours later.

Shortly thereafter, a neurological examination was performed at 39.3 h. Zack was unresponsive to noxious stimuli. The pupils remained unreactive. Corneal, oculocephalic, and gag reflex were absent. Ice water caloric testing for the vestibulo-ocular reflex was omitted because of the CSF otorrhea. An apnea test was not performed because of a bradycardia in the 44–54 range that developed at 35 h and only resolved 34 hours later when the heart rate reached 60. At 39.7 h, Zack was declared brain-dead by a certified neurologist on the grounds that he met the criteria for BD/DNC as recommended by the Guideline, according to which ancillary tests (in this case, Tc99m-DTPA scintigraphy) can confirm the diagnosis of BD/DNC when the full battery of clinical BD/DNC tests cannot be completed and/or when a confounding factor is present. A possible confounding factor in this case was the sedative effect of the propofol infusion at 60 mcg/kg/min that was continued until 38.5 h (Figure 4).

Zack's parents were informed of the BD diagnosis; they were shown the radionuclide scans and consented to organ donation as indicated on Zack's driver's license. At 40.1 h, he was put on the transplant protocol for the optimization of organs to be harvested. Levothyroxine, insulin, methylprednisolone, and vitamin K were administered between 41 and 41.7 h.

While preparations were being made for organ harvesting, a visiting cousin (a nurse by profession) grabbed Zack's right foot and scraped the sole forcefully from the heel to the toes with an unopened pocket knife around 43.5 h. Zack jerked his right foot from his cousin's hand. The cousin also jabbed his fingernail under Zack's fingernail. In response to this painful stimulus, Zack moved his arm away from the cousin, across his body. A physician, called to assess the movement as purposeful, further documented a positive corneal reflex at 43.7 h. Organ harvesting preparations were canceled. At 44.2 h, Zack was put back on the original TBI protocol, with the rate of propofol infusion increased from 60 mcg/kg/min to 65 mcg/kg/min at 47.5 h (Figure 4). Examinations by two different physicians at 48.2 h showed corneal and gag reflexes present (one physician reported that Zack grimaced and moved all extremities in response to corneal testing), oculocephalic reflex absent, pupils 3 mm and reactive bilaterally, and purposeful movement of the left upper extremity.

Zack's clinical course gradually improved. The ICP returned to normal (below 15 mmHg) by the 4^th^ day after injury. He opened his eyes spontaneously for the first time on day 7 and spoke his first words (to his parents) on day 14. He underwent tracheostomy on day 8. He was weaned off the ventilator and discharged from the ICU on day 11, able to follow commands and move all extremities. He was discharged to a rehabilitation center on day 34. On day 50 (48 days after being declared brain-dead), he left the center and returned home.

Zack also recovered cognitively such that he was able to give an interview on March 23, 2008, on NBC's “Dateline” program (Morales 2008). During the interview, Zack recounted that he remembered hearing (while comatose) a doctor say that he was brain-dead and was passing away, feeling angry about it, and being unable to communicate that he was aware.

## Critical Review of the Medical Records in 2023

The source material provided to the first author for critical review included 790 PDF pages of Zack's medical records and the image files of his radiological studies. The first task was to determine whether BD/DNC testing on Zack at 39.3 h followed the Guideline. It could be argued that it was not strictly followed because of the sedative effect of propofol which, from 30.5 h to 38.5 h was administered at the rate of 60 mcg/kg/min. Of note, however, was that after Zack was taken off the transplant protocol and put back on the original TBI protocol at 44.2 h, he received the same dose of propofol infusion (60 mcg/kg/min) until 47.5 h when the rate of infusion was increased to 65 mcg/kg/min and maintained at that level until 66.5 h (Figure 4). Despite the increasing dose of propofol, Zack showed intact corneal and gag reflexes, and purposeful movement of the left upper extremity when examined separately by two different physicians at 48.2 h. At 62.5 h, a fentanyl infusion at 25 mcg/hour was added; the rate of fentanyl infusion was increased to 50 mcg/hour at 77.5 h and remained at this level until it was discontinued 7 days later. Notwithstanding the simultaneous use of propofol and fentanyl, Zack's responsiveness steadily improved during subsequent neurological examinations. Thus, it seems unlikely that propofol could have interfered with the BD/DNC determination at 39.3 h.

Moreover, that a radionuclide scintigraphy was performed renders this issue inconsequential. According to the Guideline, an ancillary test is required when bedside neurological testing cannot be reliably evaluated due to the presence of pharmacological confounders (e.g. high dose infusion of sedatives and opioids to manage elevated ICP) and/or the presence of medical condition(s) that interfere with apnea testing or the examination of some of the cranial nerves. Zack's persistent bradycardia and CSF otorrhea precluded the testing for apnea and the vestibulo-ocular reflex, respectively. Thus, it can be safely said that BD/DNC determination followed the Guideline in Zack's case.

**Figure 5.**
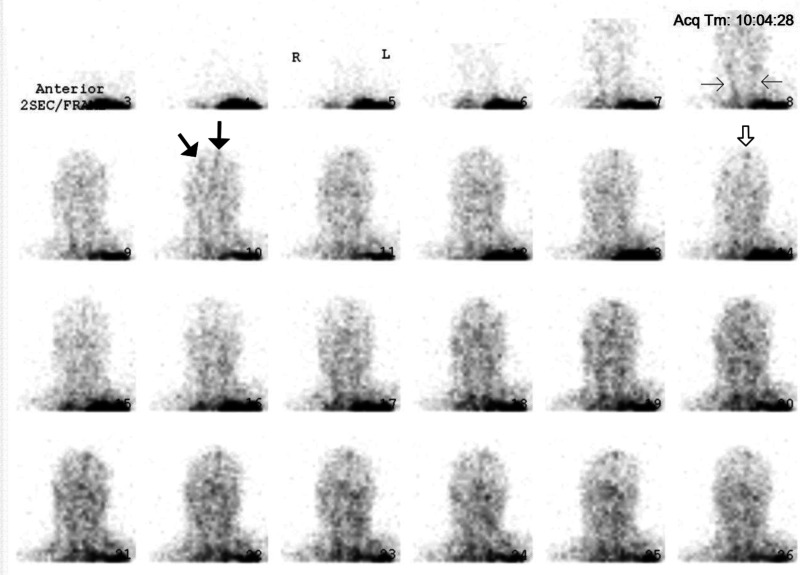
With the assistance of two of the three Nuclear Medicine (NM) experts, the subtle findings of brain blood flow become more discernible on the perfusion phase images. What seems to be “noise” in the early frames of the dynamic sequence is actually the presence of radioactivity and, therefore, a clue that flow could not have been absent. There is uptake, though faint, in the right middle cerebral artery and the paired anterior cerebral arteries, (thick arrows, frame 10). The common carotid arteries (thin arrows, frame 8) are not as well visualized as expected; they are not visible until frames 7–8. Uptake is present in the superior sagittal sinus (open arrow, frame 14).

**Figure 6.**
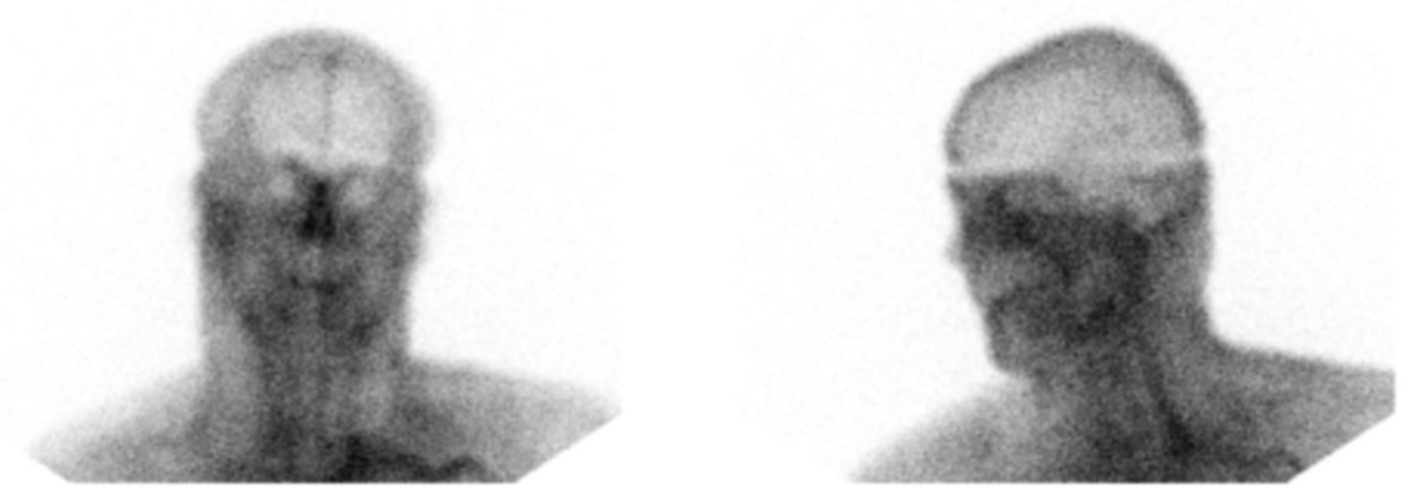
Delayed blood pool phase images showing a prominent sagittal sinus.

During the critical review of Zack's records, the first author discovered an important element in the data: his CPP was between 66 and 55 mmHg, shortly before and after Tc99m-DTPA scintigraphy, respectively. It is generally accepted that the CPP cannot drop below 60–70 mmHg for a sustained period of time without causing ischemic brain injury (Heran, Heran, and Shemie 2008, 410). Thus, Zack's CPP was in the borderline or “gray-zone” range, which suggests that BBF could have been present during the scan. The images early in the perfusion phase appeared to display more activity than usually expected; yet, the classical trident sign (formed by the right and left middle cerebral arteries together with the paired anterior cerebral arteries) indicative of the presence of BBF was not distinctly discernible (Figure 5). The superior sagittal sinus was visible on the delayed phase images (Figure 6). Since it may fill from scalp perfusion, its presence does not imply evidence of BBF, however (Zuckier and McKinnon 2023, 776). In view of these difficulties, the first author, not being a Nuclear Medicine (NM) specialist, sent the images to three internationally recognized NM experts for blind review.

According to one expert, Zack's perfusion images showed no blood flow to the cerebrum. However, this expert strongly recommended the use of Tc99m-HMPAO (a lipophilic radiotracer) in order to assess flow in the posterior fossa. According to two other experts (one of whom also preferred the use of Tc99m-HMPAO), BBF was present, but the changes were quite attenuated, with radioactivity faintly detectable in the right middle cerebral artery and the paired anterior cerebral arteries (Figure 5). The common carotid arteries were not as well visualized as expected since they did not appear in the first scans of the perfusion phase. This could explain why the cerebral arteries were poorly discernible. It was considered that the poorly visualized activity in the carotids and the cerebral arteries might have been due to some technical issue, given that scintigraphy with a lipophobic radiotracer (Tc99m-DTPA) is more operator-dependent than with lipophilics.

However, a close look at Zack's data around the time of the scintigraphy strongly suggests that the cause for the delayed filling of the carotids and the significantly attenuated signals in the cerebral arteries was none other than Zack's persistent bradycardia at the time of the procedure. It is known that poor filling of the carotids and, therefore, of the cerebrals can result from a very low systemic blood pressure or a low cardiac output. Since cardiac output is the product of heart rate and stroke volume, persistent bradycardia necessarily leads to low cardiac output and, consequently, low blood flow. While Zack was never hypotensive, he had persistent bradycardia, with a heart rate in the 44–46 range during the 2 hours before and after scintigraphy.

## Discussion

The first author's critical review of the case of Zack Dunlap brings to light that the diagnosis of BD/DNC on this patient in 2007 was incorrect. It must be admitted, however, that this was a difficult case: the critical review in 2023 could reach the correct diagnosis only with the assistance of three NM experts, of whom only two could detect the subtle presence of BBF on the Tc99m-DTPA perfusion images. A distinctive finding, in this case, is that persistent bradycardia, a contraindication for apnea testing, was most likely also the cause for the appearance of significantly attenuated BBF, such that it was misinterpreted as absent BBF. As a result of applying the Guideline algorithm, the diagnosis of BD/DNC in Zack's case hinged entirely on the result of radionuclide imaging. This case thus raises a two-fold question about the confirmatory value of scintigraphy in BD/DNC and the logic in the algorithm of the Guideline.

### Confirmatory Value of Scintigraphic Studies and the Algorithm of the Guideline

On the premise that a lack of BBF for just a few minutes causes neuronal damage, tests showing absence of BBF are deemed the most accurate ancillary investigations for BD/DNC (Young and Lee 2004, 503). The most recommended among these is BBF scintigraphy, using either lipophilic radiotracers (considered as brain-specific agents because they can cross the blood–brain barrier and be retained in the brain parenchyma) or lipophobic radiotracers. Lipophilic studies are deemed superior because the examination includes both perfusion and parenchymal phase images whereas in lipophobic studies, the perfusion phase is followed by blood pool images (Zuckier and McKinnon 2023, 775–6).

It could be argued that a limitation in Zack's case is that the radionuclide imaging in 2007 used the lipophobic Tc99m-DTPA. The 1995 and 2010 AAN Guidelines made no distinction among radiotracers, however. Furthermore, the 2003 and 2012 guidelines of the Society of Nuclear Medicine and Molecular Imaging, as well as the 2021 joint guideline by the American College of Radiology, the American College of Nuclear Medicine, the Society of Nuclear Medicine and Molecular Imaging, and the Society of Pediatric Radiology, affirm that although lipophilics are preferred, lipophobics (Tc99m-DTPA or Tc99m-pertechnetate) can be used if no lipophilic is available (American College of Radiology 2021; Donohoe et al. 2003, 2012).

Every medical test has a trade-off between sensitivity and specificity. In BD/DNC, the specificity of ancillary tests is more crucially important than sensitivity because the diagnosis of death necessarily demands 100% specificity and 0% risk of false-positive error (Shewmon 2017, 1104). According to the Guideline, the presence of BBF in patients who meet the clinical criteria of BD/DNC can be discounted as a false negative. A false positive is when BBF appears absent on radioimaging studies, yet some blood flow to at least some part of the brain is actually present. In clinical practice, such a false-positive case gets discovered only when the brain-dead patient recovers neurological functions, however partial and of short duration the recovery might be. The confirmatory value of radionuclide BBF tests therefore hinges first and foremost on their specificity. In a review on ancillary testing for BD/DNC, Young and Lee (2004, 506) laid down the following criteria for an ideal ancillary test: (i) no false positives; (ii) not confounded by drugs, metabolic disorders, or hypothermia; (iii) sufficient on its own to establish BD/DNC; and (iv) readily available, easily performed, reliable, and safe. It was concluded that radionuclide perfusion studies satisfy the aforementioned criteria which, in turn, implies a specificity of 100%. Such an affirmation necessarily presupposes that these tests had undergone rigorous validation, however.

In a review of case series of lipophilic radiotracers in planar imaging and single-photon emission CT (SPECT) between 1980 and 2008, Joffe, Lequier, and Cave (2010) estimated the sensitivity of planar imaging for clinically confirmed BD/DNC to be 119 of 153 (77.8%; 95% confidence interval [CI] 70.5%–83.7%) and the specificity to be 41 of 41 (100%; 95% CI 92.6%–100%), while the sensitivity of SPECT was 109 of 121 (90.1%; 95% CI 83.3%–94.4%) and specificity was 12 of 12 (100%; 95% CI 78.4%–100%). However, the authors indicated that all studies had a referral bias as radionuclide BBF testing was performed only when BD/DNC was strongly suspected or when bedside BD/DNC examination was incomplete or had confounding factors. They further cautioned that, given the small numbers of patients, statements about the specificity of planar and SPECT imaging are problematic, especially since these studies are performed not for prognostic purposes to indicate poor neurological outcomes or death but, rather, to diagnose the state of death. The authors concluded that the specificity of these tests should be clarified with further studies.

In a recent meta-analysis of cohort and case studies on BD/DNC ancillary tests found in the literature from its inception to February 4, 2022, Briard et al. (2023) reported that the pooled specificity estimates for lipophobic (Tc99m-DTPA) and lipophilic radionuclide studies, which together comprised 16% of ancillary tests, were 100% (458/458). The authors cautioned, however, that for all types of ancillary tests, most estimates had high statistical uncertainty because 99% of the eligible studies demonstrated a high risk of bias on at least one QUADAS-2 (Quality Assessment of Diagnostic Accuracy Studies-2) domain. Of note, only 7% of the studies had a low risk of bias for patient selection. The authors therefore concluded that further high-quality studies are required for a rigorous validation of all ancillary tests, including BBF scintigraphy.

The reviews of both Joffe, Lequier, and Cave (2010) and Briard et al. (2023) bring to the fore the fact that, while radionuclide BBF tests are widely used, the question about their specificity has not been adequately addressed. A fundamental problem, not mentioned by these authors, is the issue of the reference “gold standard.” In all the aforementioned studies on the specificity of radionuclide tests, the bedside clinical criteria of BD were used as the reference “gold standard” even though they have never undergone rigorous validation.

As confirmed by the 2008 Canadian Expert Consensus Meeting on Brain Blood Flow in the Neurological Determination of Death, none of the current ancillary tests for BD/DNC, including those accepted as “gold standards” (namely, four-vessel angiography and radionuclide imaging), have undergone rigorous evaluation to demonstrate that they have an infinitesimally small false-positive rate (Shemie et al. 2008, 144). According to NM scholars, validation studies to define the specificity of scintigraphy for the determination of BD must include a large cohort of nonbrain-dead patients, that is, patients with catastrophic brain injury but without complete loss of neurological function (Zuckier 2022, 1324). Such an evaluation remains to be undertaken because currently, ancillary tests are simply not performed in this group of patients. Consequently, without rigorous validation, the specificity of radionuclide imaging remains undetermined and the rate of false positives is unknown. However, since it is a test for the determination of death, a single documented false positive is sufficient to indicate that the unknown rate is at least known not to be infinitesimal, as ethics requires it must be.

In a series of 219 consecutive patients with lipophilic Tc99m-pertechnetate studies, no detectable BBF was reported in five of 10 nonbrain-dead patients, of whom two had persistent reflexes and three had minimal respiratory activity (Flowers and Patel 1997). In addition, three case reports of patients who were brain-dead (minus apnea testing) have shown that BBF scintigraphy, whether lipophobic or lipophilic, lacks the required 100% specificity necessary for establishing BD/DNC. In the first case, radioimaging using the lipophilic Tc99m-ethyl-cysteinate-dimer performed on a 9-month-old infant, in whom apnea testing was omitted due to acute respiratory distress syndrome, showed no detectable radiotracer activity on the perfusion- and parenchymal-phase images. Upon life support removal, however, the infant had breathing efforts for over 20 minutes until her death by circulatory criteria (Joffe, Lequier, and Cave 2010). In the second case, a Tc99m-DTPA scintigraphy performed on a 2-year-old boy, in whom apnea testing was aborted because of oxygen desaturation, showed absent BBF. Following the declaration of death and the withdrawal of the ventilator, he began to breathe spontaneously. He was reconnected to the ventilator. Because of the grim prognosis, it was agreed with family members to withdraw the ventilator a second time. He remained apneic thereafter until cardiac arrest (Shewmon 2017). In the third case, a lipophilic Tc99m-bicisate SPECT study performed on a 59-year-old man, in whom apnea testing was omitted due to hemodynamic instability requiring multiple vasopressors, showed no radiotracer activity in the cerebral hemisphere and posterior fossa. Death was declared and the family consented to organ donation. The following morning, however, he had a cough reflex, intermittent spontaneous respirations, and extensor posturing of the right arm and leg upon noxious stimulation. He subsequently developed a generalized seizure leading to ventricular tachycardia and cardiac arrest (Latorre, Schmidt, and Greer 2020). In all three cases, BBF was definitely not present on the scans. In Zack's case, BBF was present but at such a diminished level that radiotracer activity in the cerebral arteries could be identified by only two of three experts in NM during the critical review of the case in 2023, thus revealing that the scan was misread in 2007, though understandably, forgivably, and likely to have been similarly misread by many other NM interpreters at other institutions. This fact would have never come to light if Zack had undergone organ removal following the declaration of death based on the diagnosis of BD/DNC, which he was literally minutes away from and which would have occurred if a nurse-cousin had not been present and independently tested again for responsiveness to noxious stimuli.

It may be said that Zack's scans were in the “gray zone,” that is, the gray area in the continuum between unequivocal flow and unequivocal no-flow. The result is a pseudo-false positive which, nevertheless, led to an incorrect diagnosis of BD/DNC. To the best of the authors’ knowledge, this type of “gray zone” case (in which the diagnosis of BD/DNC hinges on BBF scintigraphy because clinical assessment was not fully complete due to safety reasons) has not been reported. Zack's case brings into focus that the difference in appearance between no flow and low flow can be quite subtle, which in turn makes it perilous to rely on radionuclide scans for establishing the diagnosis of BD/DNC on patients in whom the clinical assessment cannot be safely or fully completed. Since level II regional trauma centers in the United States are not necessarily staffed with NM experts, it is not implausible that other instances of misinterpretation similar to Zack's case might have occurred over the 50-some years that radionuclide scintigraphy has been in use as an ancillary test throughout the world. There is no way to document such occurrences, however rare they might be since they lead to a diagnosis of BD/DNC, following which the patient is either removed from life support or destined for organ harvesting.

All the aforementioned cases, including the current case, point to a fundamental problem in BBF scintigraphy, namely, that studies to determine thresholds of minimal detectable perfusion have never been performed for either lipophobics or lipophilics despite the fact that these parameters are essential to the interpretation of BBF scintigraphy (Zuckier 2022, 1324). That such studies might not be readily feasible could be because direct measurements of BBF (expressed in units of milliliters of blood per 100 grams of brain tissue per minute) require experimental or investigational tools such as positron emission tomography. Since BBF is the ratio of CPP to cerebrovascular resistance (CVR), and CPP is the difference between MAP and ICP, the calculation of CPP may be used as a surrogate for BBF in the clinical setting (Fantini et al. 2016; White and Venkatesh 2008, 979), but only if CVR remains relatively constant. Multiple studies have shown that cerebral autoregulation (which controls CVR) is impaired in many patients with TBI, however, making it difficult to derive a relationship between CPP and BBF (White and Venkatesh 2008, 981). Furthermore, accurate calculation of CPP requires that the measurement points for both MAP and ICP be at the same level, that is, at the level of the midbrain using the tragus of the ear as an external landmark (Smith 2015, 489). A survey of 309 European neurosurgical centers showed that, in many hospitals, 30-degree head elevation is used in patients with severe TBI, but MAP is calibrated at the heart level. In such conditions, and taking into consideration the 30-cm distance between the heart and the head, the CPP reading would be 11 mmHg higher than what it truly is (Rao et al. 2013). In the current case, though Zack had both MAP and ICP monitoring, BBF levels could not be estimated since both the status of his cerebral autoregulation and the information about MAP calibration remained unknown.

Irrespective of the type of radiotracers (lipophobic or lipophilic), false positives (whether true or pseudo-) in BBF scintigraphy are concerning as they can lead to serious bioethical consequences, namely, the declaration of death through the application of the algorithm of the Guideline. On the one hand, the Guideline considers ancillary BBF studies noncontributory in confirming BD/DNC when the diagnosis is achieved based on the complete battery of bedside neurological tests, including apnea testing. The reasoning in this arm of the algorithm is that ancillary BBF studies are not free of false negatives, that is, the patient fulfills the clinical criteria of BD/DNC but the ancillary study shows evidence of BBF. Such reasoning contains two presuppositions: (i) the clinical criteria stand as the reference point (a “gold standard” as it were), which in turn implies that bedside neurological tests are foolproof despite the fact that they have never been validated, and (ii) the clinical tests are performed by skillful specialists in neurosciences (Wijdicks 2010, 81). On the other hand, the Guideline (especially the 1995 Guideline that was applicable to Zack's case in 2007) stipulated that ancillary testing is necessary for establishing the diagnosis of BD/DNC when specific components of clinical testing cannot be reliably evaluated. The logic in this second arm of the algorithm is problematic because if an ancillary BBF study (namely, BBF scintigraphy) is deemed useless in confirming the clinical diagnosis of BD/DNC due to false negatives, in what way can it become a declarative test to establish the diagnosis of BD/DNC when it is not free of false positives? In other words, given that a single false-positive diagnosis of death is one too many, the second arm of the Guideline algorithm cannot be applied unless the ancillary BBF scintigraphy possesses an essentially zero false-positive rate, which it does not, however.

In addition, there is another reason why the second arm of the algorithm cannot be applied. As shown in Zack's case, and most likely also in the aforementioned case reported by Latorre, Schmidt, and Greer (2020) cardiovascular disturbances which preclude apnea testing are also the very conditions which can cause a temporary drop in BBF to a level below (or around) the detectability of scintigraphic studies, such that radiotracer activity is not visible (or so faintly present that it is missed) on the scans. In other words, conditions such as bradycardia or severe hypotension are contraindications to apnea testing and, according to the second arm of the algorithm, necessitate the use of BBF scintigraphy. Yet, these conditions are precisely those which can result in false positives (whether true or pseudo-) in BBF radionuclide studies.

### Cognitive-Motor Dissociation

An unexpected finding in the present case is that, according to Zack's account, he heard a doctor telling his parents that he was brain-dead and was passing away; he also remembered feeling angry about it as he could not make known that he was aware. Around the time of this incident when Zack was declared brain-dead, he was comatose and completely unresponsive by physical examination. His CPP was 55 mmHg. It is unclear whether this CPP level could have corresponded to a BBF level in the penumbral range (i.e. a BBF level insufficient to support function but still adequate enough to maintain viability), between 10–15 mL/100gm/min and 25–35 mL/100gm/min (Fisher and Bastan 2012, S79). It is clear, however, that what took place in Zack is similar to what has been reported as intraoperative awareness with explicit recall during general anesthesia (Mashour, Orser, and Avidan 2011; Phillips et al. 1993; Sebel et al. 2004) which occurs at an incidence of 0.1–0.2%, irrespective of potential differences in anesthetic management. The common characteristic feature between Zack's case and patients who had intraoperative awareness with explicit recall is dissociation between arousal (wakefulness) and awareness.

The meaning of the term “consciousness” can vary among various scholars. For some, consciousness strictly refers to awareness (Crick and Koch 1990; Tononi and Koch 2015). For most scholars, consciousness refers to both arousal and awareness, that is, the level and the experiential content of consciousness, respectively (Edlow et al. 2017; Laureys 2005; Mashour, Orser, and Avidan 2011; Zeman 2001). Arousal refers to the level of alertness which encompasses the whole spectrum of waking states, ranging from wakefulness to coma. The degree of arousal can be assessed by a third-party observer using objective criteria such as the GCS to document the patient's motor or verbal responses to various stimuli, including noxious stimuli. Unlike arousal, awareness is a qualitative, intimately private, and subjective first-person experience ranging from perceptual awareness of the environment to self-awareness. As such, awareness is inaccessible to observation by third parties during clinical examination (Zeman 2006), unless the subject manifests a behavioral response that is nonreflexive and can be considered as volitional.

In the literature on BD/DNC, it has been claimed that: (i) one must be awake in order to be aware, and that (ii) conversely, patients in coma are unconscious (i.e. with neither wakefulness nor awareness) because they are unresponsive or cannot be awakened (Bernat 2009, 382). However, such claims overlook the fact that wakefulness and awareness do not always run in parallel (Zeman 2006, 359), as seen in cases of intraoperative awareness with explicit recall during general anesthesia (Mashour, Orser, and Avidan 2011; Phillips et al. 1993; Sebel et al. 2004). The question at hand is whether disconnection between arousal and awareness can occur in coma.

A landmark study in 2006 demonstrated that the lack of a motor or verbal response does not necessarily imply a lack of awareness. This was shown in a patient with unresponsive wakefulness syndrome (UWS) who nevertheless was able to follow verbal instructions to perform two mental imagery tasks during a functional magnetic resonance imaging (fMRI) scan (Owen et al. 2006). Thereafter, several studies have shown that a small number of patients clinically diagnosed with UWS demonstrated brain activation on fMRI or electroencephalogram (EEG) in response to spoken commands—a finding indicative of residual cognitive function and awareness in these patients (Cruse et al. 2011; Monti et al. 2010). This phenomenon, observed in some severely brain-injured patients, is now referred to as “CMD” to describe the sharp dissociation between a retained but unrecognized cognitive capacity and the absence of purposeful behavioral responses (Schiff 2015, 1413). Cognitive-motor dissociation is also referred to as “covert consciousness,” a terminology which underscores the fact that preserved awareness in patients who appear unresponsive evades bedside neurological examination, and its detection necessitates the use of advanced diagnostic tools (namely, functional neuroimaging and task-based electrophysiological techniques) which currently are not fully implemented in clinical practice (Young, Edlow, and Bodien 2024).

The phenomenon of CMD is found not just in patients with UWS but also in comatose patients. In a prospective study using task-oriented EEG on 104 patients with acute brain injury among whom 56 were in coma, CMD was found in 16 patients, among whom eight were comatose (Claassen et al. 2019). These patients, though behaviorally unresponsive, manifested EEG responses to motor commands, thus showing that they were aware. It can be said that Zack's condition around the time of his BD/DNC determination was closely similar to this group of comatose patients with CMD. Though he was completely unresponsive, he was aware of his surroundings and his own feelings when hearing what was being said about him. If a task-oriented EEG or fMRI had been performed on Zack around that time, it is not inconceivable that brain activation would have been detectable by either technique, which then would have proved that Zack was not brain-dead. It has been cautioned, however, that the absence of fMRI and EEG responses, that is, the absence of evidence of covert consciousness, does not constitute evidence of the absence of covert consciousness (Edlow et al. 2017, 2412; Young, Edlow, and Bodien 2024, 33).

As repeatedly pointed out by various authors, the occurrence of CMD in severely brain-injured patients indicates that brain function, especially consciousness, cannot be adequately evaluated by bedside clinical examination alone (Claassen et al. 2019; Cruse et al. 2011; Edlow et al. 2017; Monti et al. 2010; Rohaut, Eliseyev, and Claassen 2019; Young, Edlow, and Bodien 2024). In this light, it is rather disconcerting that the 2020 World Brain Death Project (Greer et al. 2020), the 2023 Canadian Clinical Practice Guideline (Shemie et al. 2023), and the AAN Guideline emphasize that BD/DNC is a clinical diagnosis made at the bedside based on the triad of unresponsive coma with absence of the capacity for consciousness, absence of brainstem reflexes, and absence of the capacity to breathe independently. The Canadian guideline even explicitly defines absence of consciousness as the “lack of wakefulness and awareness in response to stimuli” (Shemie et al. 2023, 485). However, as discussed above, unresponsiveness cannot be equated with absence of consciousness since some unresponsive comatose patients can still manifest awareness. In fact, the triad of motor unresponsiveness to external stimuli, brainstem areflexia, and apnea has never been validated to exclude the capacity for conscious awareness in humans (Rady 2021, 1576). In other words, large-scale prospective studies are urgently needed to establish whether or not CMD occurs in patients who fulfill the criteria of BD/DNC. Without addressing this important issue, the validity of the BD/DNC paradigm will be called into question.

## Conclusion

It has been asserted repeatedly by AAN scholars that with the compliant application of the Guideline, there has been no false-positive determination of death (Lewis et al. 2018, 536; Russell et al. 2019, 229; Wijdicks et al. 2010, 1912). However, several published cases (including the three cases cited in this paper) have falsified such assertions (Shewmon 2023), especially the extraordinary case of a false-positive BD diagnosis in Jahi McMath, in which not only all the clinical criteria of BD were fulfilled but four EEGs and a BBF radionuclide test also showed absent brain electrical activity and absent BBF, respectively (Shewmon and Salamon 2021). Unlike these cases, Zack's case is a pseudo-false positive in which there were several factors at play, including: (i) application of the second arm of the Guideline algorithm; (ii) Zack's persistent bradycardia; (iii) low BBF in the “gray zone;” (iv) lack of an experienced NM expert on-site to make a correct interpretation of the difficult scans; (v) no existing validation studies establishing the thresholds for BBF radionuclide tests; and (vi) the specificity of these tests, irrespective of whether the radiotracer is lipophilic or lipophobic, is not 100%. The first and last are the most critical factors because it defies logical reasoning that an ancillary test without a specificity of 100% (i.e. zero risk of false positive) is recommended by the Guideline to establish the diagnosis of BD in cases with incomplete clinical evaluation. The only solution to avoid the risk of a false-positive (whether true or pseudo-) BD/DNC diagnosis in such instances is not to apply the second arm of the Guideline algorithm, especially when the indications for its application are instances in which the patients manifest hemodynamic or cardiac instability. In other words, in such instances, it is preferable to adopt the more prudent approach of “watch and wait” to see how the patient's clinical course unfolds.

The grave bioethical consequences of a false-positive declaration of death based on a false-positive (whether true or pseudo-) BD/DNC diagnosis are self-evident. In previously reported cases of false-positive BD/DNC diagnoses, the patient either succumbed to circulatory-cardiac-respiratory death shortly after the return of brain function following the declaration of BD/DNC or, as in Jahi's case, continued to survive with severe neurological debilitation until the occurrence of circulatory-cardiac-respiratory death. That all these patients had a grim neurological prognosis does not mitigate the gravity of a false-positive declaration of death, however. In Zack's case, the gravity of a false-positive declaration of death is greatly magnified because, had he not narrowly “escaped” organ harvesting, he would not be alive today, holding steady employment and raising a family.
